
JOURNAL INFORMATION
==============================
NLM Title Abbreviation: ScientificWorldJournal
Journal ID: ScientificWorldJournal
NLM Journal ID: 101131163
Journal ID: TSWJ

The Scientific World Journal

ISSN: 2356-6140
EISSN: 1537-744X
Publisher: Wiley

ARTICLE INFORMATION
==============================
PMCID: PMC6084141
PMID: 30147587
DOI: 10.1100/tsw.2001.124
Article ID: 867920
Article version: 1
Subjects: Short Report

Stimulation of Apoptosis by Computationally Derived Small Molecules that Bind to BCL-2

Mutomba Martha *
Wang Jing
Mailiartchouk Sergei
Brady Tom
Rideout Darryl
Niemeyer Christina
Zhu Hengyi
Fisher Cindy
Mong Seymour
Ramnarayan Kal
Structural Bioinformatics Inc., 10929 Technology Place, San Diego, CA 92127, USA
*Martha Mutomba: mutomba@strubix.com
Publication date: 2001
Electronic publication date: 2001 Dec 18
Volume: 1
First page: 107
Last page: 107
Copyright: Copyright © 2001 Martha Mutomba et al.
Copyright year: 2001
License: This is an open access article distributed under the Creative Commons Attribution License, which permits unrestricted use, distribution, and reproduction in any medium, provided the original work is properly cited.
License URL: https://creativecommons.org/licenses/by/3.0/

Funding: National Institutes of Health

==============================
Miami *Nature Biotechnology* Short Reports
*TheScientificWorld* (2001) 1(S3), 107SR
ISSN 1532-2246; DOI 10.1100/TSW.2001.124

### STIMULATION OF APOPTOSIS BY COMPUTATIONALLY DERIVED SMALL MOLECULES THAT BIND TO BCL-2

Martha Mutomba*, Jing Wang, Sergei Mailiartchouk, Tom Brady, Darryl Rideout, Christina Niemeyer, Hengyi Zhu, Cindy Fisher, Seymour Mong, and Kal Ramnarayan

Structural Bioinformatics Inc., 10929 Technology Place, San Diego, CA 92127, USA
* mutomba@strubix.com

**INTRODUCTION.** Bcl-2 and its family members are key regulators of apoptosis or programmed cell death, implicated in human diseases including cancer (1). The family has both anti-apoptotic members such as Bcl-2 and Bcl-$x_L$ and also death-promoting members such as Bax, Bak, Bid and Bad (1,2). The interactions between these two groups of proteins have been shown to modulate the sensitivity of a cell to apoptotic stimuli (3,4). Most cancers typically show high levels of Bcl-2 expression, and this overexpression is believed to contribute to the resistance of cancers to a wide variety of chemotherapeutic drugs and radiation therapy (5). In our studies, we used structure-based computer screening technology to identify small molecule Bcl-2 antagonists. These compounds were assayed for binding to Bcl-2 and then in cell-based functional assays. Positive compounds can potentially be developed as anti-cancer therapies.

**METHODS.** The NMR structure of Bcl-$x_L$ complexed with a portion of the BH3 region of its ligand Bak (PDB accession number 1BXL) (6) was used to generate a computational model of Bcl-2 complexed with a corresponding portion of its intracellular ligand Bax. The model was used in a 3-D computational search for non-peptide small molecules that disrupt Bcl-2/Bax interaction. Selected compounds were tested for their ability to inhibit binding of $Eu^{3+}$-labelled Bax to Bcl-2 as measured by time-resolved fluorometry. Compounds with Ki values less than 50 μM were tested for their ability to induce apoptosis in Jurkat cells that overexpress Bcl-2 (Jurkat/Bcl-2 cells). The published Bcl-2 binding compound HA14-1 (7), was used as a control in the biochemical and cellular assays.

**RESULTS.** Two compounds with Ki values of 6 μM and 25 μM in the binding assay showed anti-Bcl-2 activities in cell-based assays. Jurkat/Bcl-2 cells are resistant to apoptosis induced by 1 μM staurosporine, as measured by cleavage of caspase 3 and PARP. However, caspase 3 and PARP cleavage were observed when these cells were incubated with 1 μM staurosporine in addition to either of these two compounds at 30 and 100 μM. Furthermore, the compounds induced apoptosis in a breast cancer cell line, MCF-7, which overexpresses Bcl-2.

**CONCLUSION.** Two chemically distinct compounds were identified as Bcl-2 antagonists, leading to induction of apoptosis in Bcl-2 overexpressing cells. These results suggest that targeting Bcl-2 may lead to the development of new anti-cancer agents.

**ACKNOWLEDGEMENTS.** This work was supported in part by a grant from NIH.

**REFERENCES.**

1. Chao, D. and Korsmeyer, S.J. (1998) Annu. Rev. Immunol. 16, 395-419
2. Adams, J. and Cory, S. (1998) Science 281, 1322-1326
3. Yin, X.-M., Oltvai, Z. N., and Korsmeyer, S.J. (1994) Nature 369, 321-323
4. Oltvai, Z.N. and Korsmeyer, S.J. (1994) Cell 79, 189-192
5. Reed, J.C. (1999) Curr. Opin. Oncol. 1, 68-75
6. Sattler, M., Liang, H., Nettsheim, D., Meadows, R.P., Harlan, J.E., Eberstadt, M., Yoon, H.S., Shuker, S.B., Chang, B.S., Minn, A.J., et al. (1997) Science 275, 983-986
7. Wang, J.-L., Liu, D., Zhang, Z.-J., Shan, S., Han, X., Srinivasula, M., Croce, C.M., Alnemri, E.S., and Huang, Z. (2000) Proc. Natl. Acad. Sci. U S A 97, 7124-7129
